# Kinematic Characteristics of the Racket in Table Tennis During Backhand Flick Followed by Backhand Fast Block and Forehand Fast Block Combinations

**DOI:** 10.3390/s26092818

**Published:** 2026-04-30

**Authors:** Jianfeng Niu, Chingleong Gan, Xinyu May Teo, Yaqi Xue, Xiaojie Guo, Wenlong Ma, Haobai Li, Zhikun Gao, Zhiping Zhen

**Affiliations:** 1 Physical Education of Sport Training, Beijing Sport University, Beijing 100084, China; 2245@bsu.edu.cn (J.N.);; 2 College of Physical Education and Sport, Beijing Normal University, Beijing 100875, China; 202539070001@mail.bnu.edu.cn (C.G.); xueyaqi555@mail.bnu.edu.cn (Y.X.); 3 Department of Physical Education, Peking University, Beijing 100871, China; 2401212220@stu.pku.edu.cn; 4 Department of Physical Education, Nankai University, Tianjin 300071, China; 5 School of Physical Education, Shanghai University of Sport, Shanghai 200438, China

**Keywords:** kinematic analysis, technical combination, performance optimization, table tennis, racket

## Abstract

A backhand flick is frequently used in table tennis to initiate offensive play, yet how racket motion evolves during subsequent stroke transitions remains insufficiently characterized. This study examined racket kinematics in two common follow-up combinations: a backhand flick followed by a backhand fast block (BFBB) and a backhand flick followed by a forehand fast block (BFFB). In a within-subject design, ten national-level male players performed both combinations, and racket motion was recorded using a three-dimensional motion capture system at 200 Hz. Racket velocity, phase duration, and spatial displacement were quantified across the stroke sequence, and within-player differences between the two stroke transition combinations following the backhand flick were examined. Compared with BFBB, BFFB showed higher racket velocity at most key moments, particularly near ball contact, whereas no significant difference was found at the end of the follow-through. Backward-phase duration did not differ between the two conditions, but BFFB showed longer durations during the hitting and follow-through phases, together with a longer overall duration. BFFB also exhibited greater directional displacement across multiple phases, whereas BFBB was characterized by a more compact spatiotemporal pattern. These findings provide biomechanical evidence that different follow-up strokes after an identical backhand flick are associated with distinct patterns of racket motion during stroke transitions and may offer a kinematic reference for sequence-specific training in table tennis.

## 1. Introduction

Table tennis is a high-speed racket sport in which players must respond to the ball within extremely short time intervals. The ball travels fast, the playing distance is limited, and rallies unfold rapidly, leaving little time for adjustment [1]. Players must rapidly read the opponent’s movement and the incoming ball trajectory, select an appropriate response, and organize stroke execution within a highly constrained time window [2]. In match play, perceptual processing, response selection, and motor execution are closely coupled rather than fully separable processes [3]. This close coupling of visual input and motor output places substantial demands on the sensorimotor system and is supported by evidence that table tennis involves coordinated neural processing across visual, motor, and executive systems [4,5]. At higher levels of play, performance depends not only on physical capacity but also on the ability to maintain movement organization under severe temporal constraints [6,7]. Long-term training has been shown to improve motor coordination and control, supporting the execution of complex stroke sequences when preparation time is limited [8,9]. From a technical standpoint, this ability is reflected in how efficiently players control racket motion, regulate stroke timing, and adapt movement organization across successive actions during play [10,11]. Neurophysiological evidence further indicates that experienced players develop more stable visuomotor control patterns, which may contribute to greater consistency and precision in stroke execution [5,12].

In recent years, changes in equipment have further influenced how the game is played [13]. The introduction of the 40+ mm plastic ball has altered both the technical and tactical characteristics of table tennis [14,15]. Compared with the former celluloid ball, the plastic ball produces less spin and slightly reduced ball speed [15]. As a result, controlling the early phase of the rally has become increasingly important, particularly during serve and return exchanges within the so-called “first three strokes” [16]. In this context, the backhand “banana flick” (also known as the Chiquita) has become a strategically essential technique. It allows players to aggressively return short serves using high sidespin and forward acceleration, even under spatial and temporal constraints [17]. Despite its widespread use at the elite level, the biomechanical features of the banana flick, especially how it links into subsequent strokes, have not yet been examined in sufficient detail.

Recent advances in motion capture (MoCap) technologies have made it increasingly feasible to examine the biomechanics of rapid, sport-specific movements in greater detail. High-speed optical systems and wearable inertial measurement units (IMUs) now allow three-dimensional tracking of athletes and equipment at sampling rates exceeding 200 Hz, making it possible to quantify joint angles, segmental velocities, and racket trajectories with millisecond-level resolution [18,19]. MoCap has been widely used to analyze kinematic parameters in racket sports such as badminton and tennis, and its application in table tennis has yielded insights into forehand topspin and service motions [20,21]. To date, relatively few studies have used Mo Cap techniques to examine complex transition phases within multi-stroke sequences, including those following the backhand banana flick.

Systematic reviews of table tennis biomechanics indicate that existing research has largely concentrated on isolated strokes and elite male athletes, with comparatively less attention given to multi-stroke dynamics, inter-stroke timing, and sex-specific motor adaptations [22,23]. While prior studies have successfully described the joint kinetics and segmental coordination of isolated forehand strokes, the biomechanical complexity of transitions between consecutive actions has received far less attention. This is particularly true for stroke sequences involving the backhand banana flick and its immediate follow-up, where rapid redirection and minimal preparatory motion are required [24]. This gap in empirical data forces coaches to rely heavily on qualitative judgment when designing training interventions and limits their ability to assess performance based on quantitative markers such as racket displacement, velocity peaks, or recovery timing [25].

The present study focused on two backhand-initiated stroke combinations in table tennis: a banana flick followed by either a backhand fast block or a forehand fast block [16]. Racket kinematics were examined in terms of three-dimensional displacement, velocity, and temporal characteristics, with particular attention to how racket motion was organized during stroke transitions in fast exchanges [10,26]. The aim was to clarify the racket-level kinematic characteristics of these follow-up techniques and to provide a quantitative reference for understanding post-flick stroke-transition combinations.

## 2. Materials and Methods

### 2.1. Participants

Ten male table tennis players classified as Chinese National Level I athletes participated in this study. All were right-handed, shakehand-grip players enrolled in the Table Tennis Sport Training Program at Beijing Sport University. Participant characteristics, including age, training experience, height, body mass, and skill level (Table 1). None had a history of upper or lower limb injuries or surgeries. All participants were informed of the study procedures and provided written informed consent before data collection.

A post hoc power analysis was performed in G*Power 3.1 for the paired-samples *t*-test used in this within-subject design (two-tailed, α = 0.05). Based on the observed effect size of the primary outcome (dz = 1.86) and a sample size of 10, the achieved statistical power was 0.999, which should be interpreted considering the large observed effect and the within-subject nature of the study.

### 2.2. Experimental Setup and Procedure

Prior to data collection, participants’ anthropometric measurements (i.e., height and weight) were obtained using a calibrated stadiometer and electronic scale. Kinematic data were recorded using an eight-camera Qualisys motion capture system (Arqus A12, Qualisys AB, Gothenburg, Sweden) operating at 200 Hz. Reflective markers were affixed to the racket to enable three-dimensional tracking of its movement trajectories. Camera placement was optimized to ensure full capture of racket motion during stroke execution. Each camera’s height, tilt, and focal length were adjusted to minimize marker occlusion and projection distortion. Cameras were positioned approximately 3 m from the movement center, and the primary lighting source was set 0.8 m above ground. System calibration was performed using a wand equipped with reflective markers, which was moved throughout the capture volume for 10 s (1000 frames). Calibration parameters were computed using Qualisys Track Manager software (Version 2026.1) and verified for spatial accuracy.

Before formal testing, participants were introduced to the laboratory environment and testing equipment. A standardized 20 min warm-up was completed, consisting of dynamic stretching and a 10 min multiball practice session. During this period, participants practiced the two stroke combinations examined in the study, namely a backhand flick followed by either a backhand fast block or a forehand fast block.

**Figure 1:**
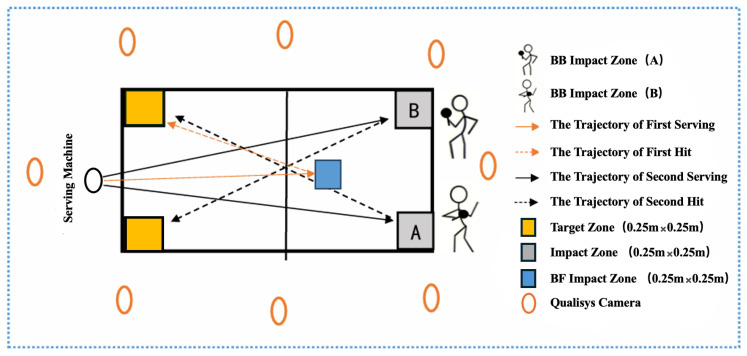
Schematic representation of the experimental setup. The figure illustrates the experimental layout for analyzing two-stroke combinations: a backhand flick followed by either a backhand fast block (BFBB) or a forehand fast block (BFFB). The serving machine delivered the first ball to the BF impact zone (blue square), where the player executed a backhand flick toward the target zone on the opposite side of the table (yellow square). The second ball was then delivered to impact area A or B (gray squares), where the player performed the corresponding follow-up stroke toward the designated target zone. Colored arrows indicate ball trajectories, and eight Qualisys cameras (orange ovals) surrounded the setup to capture three-dimensional racket motion during each phase.

Testing was conducted at the Table Tennis Training Center of Beijing Sport University on a professional table tennis table (Rainbow, Double Happiness Sports Company, Shanghai, China). Two impact areas and two target areas (0.25 m × 0.25 m) were marked on the table. Participants executed the initial backhand flick in impact area A, moved to impact area B using a chasse step, and then performed the follow-up stroke toward the corresponding target area (Figure 1). The testing order was fixed across participants, with a 3 min rest interval between the two stroke combinations. Balls were delivered by a PongBot table tennis robot (model NOVA), and feeding parameters were kept constant across all trials and conditions. The same type of ball was used throughout testing (Double Happiness three-star D40+ ABS). Participants wore table tennis shoes, and all trials were performed on the same playing surface. Each stroke combination was tested in five valid trials, and each session was completed within approximately 40 min. A trial was considered valid when the prescribed stroke sequence was completed correctly, and the ball landed in the designated target area. No trials were excluded, and the three best trials for each condition were retained for analysis. Fatigue was considered unlikely to have materially influenced performance because the number of repetitions was limited and rest was provided between conditions.

### 2.3. Definition of the Motion Phase

**Figure 2:**
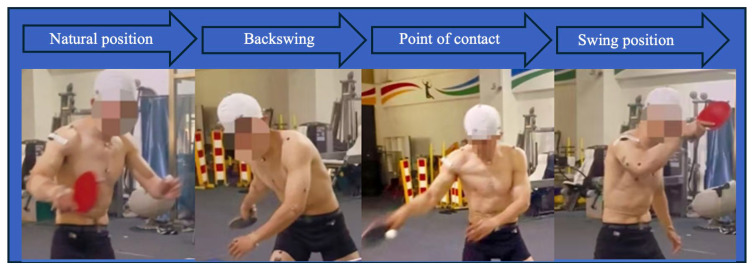
Starting from the natural position until the swing position for the backhand fast block.

**Figure 3:**
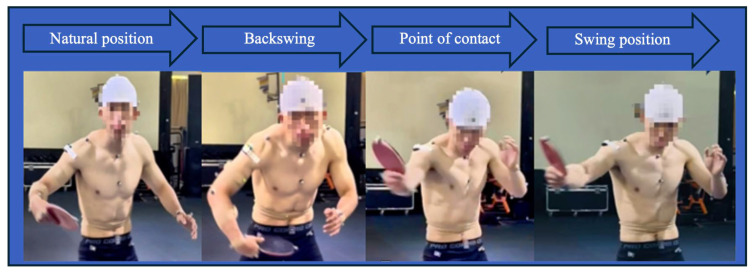
Starting from the natural position until the swing position for the forehand fast block.

The temporal segmentation of the stroke sequence, including the backhand flick followed by a backhand fast block (BFBB) or forehand fast block (BFFB), is illustrated in Figure 2 and Figure 3. Point A was defined as the natural position (NP). The interval from A to B represented the backward phase (BP). The hitting phase (HP) was defined from point B to C, which encompassed the moment of ball contact. The follow-through phase (FP) extended from C to D, with point D corresponding to the end of swing position (PE).

### 2.4. Data Processing

Kinematic analyses were performed using the Qualisys motion capture data. Each stroke cycle was defined as the interval from the end of one follow-through to the completion of the next. Racket motion was divided into three phases: backswing, hitting, and follow-through. Temporal variables were expressed in seconds (s), displacement variables in meters (m), and velocity variables in meters per second (m/s). The analysis focused on racket movement time, racket velocity, and spatial displacement under the BFBB and BFFB conditions.

### 2.5. Statistical Analysis

Statistical analyses were conducted using IBM SPSS Statistics (version 29.0; IBM Corp., Armonk, NY, USA). All variables are presented as mean ± standard deviation (SD). For each participant, repeated trials performed under each stroke combination (BFBB and BFFB) were first averaged, and participant-level mean values were subsequently used for statistical comparisons. Racket velocity was quantified at six predefined characteristic moments of the stroke (NP, BE, BV, CP, VM, and PE). Temporal parameters included the durations of individual stroke phases (BP, HP, FP, and EP), as well as the combined HP + FP phase. Racket movement trajectories were described using phase-specific displacements along the left–right (LR), forward–backward (FB), and up–down (UD) axes during the BP, HP, FP, and TD phases. The normality of paired differences between BFBB and BFFB was examined using the Shapiro–Wilk test, and all variables satisfied the normality assumption. Paired-samples *t*-tests were used to compare racket velocity, phase duration, and three-dimensional displacement measures between the two stroke combinations. To control for multiple comparisons, the Holm–Bonferroni correction was applied within each family of related tests. For each comparison, 95% confidence intervals (CIs) and Cohen’s dz were also reported. Statistical significance was set at α = 0.05.

## 3. Results

### 3.1. Motion Velocity Characteristics of Racket

**Figure 4:**
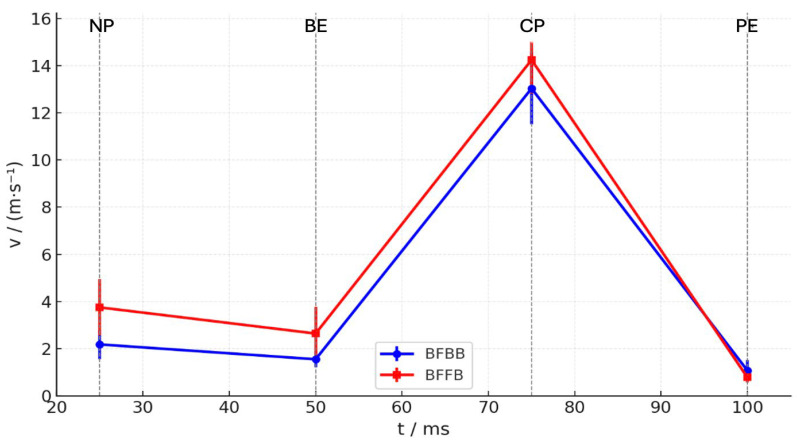
Comparison of resultant racket velocity at NP, BE, CP, and PE between BFBB and BFFB.

Table 2 and Figure 4 show the resultant racket velocity at the characteristic moment of the stroke cycle, including the natural position (NP), end of the backswing (BE), velocity maximum of backswing (BV), point of contact (CP), velocity maximum (VM), and end of the swing position (PE). Differences between BFBB and BFFB were observed at several moments of the stroke sequence. At NP and BE, racket velocity was higher in BFFB than in BFBB (NP, *p* < 0.001; BE, *p* < 0.01). During the acceleration phase toward ball contact, velocity increased in both stroke combinations, with higher values observed in BFFB at BV, CP, and VM (all *p* < 0.001). At PE, velocity had decreased in both conditions, and no significant difference was observed between BFBB and BFFB (*p* = 0.079). Both stroke combinations showed a similar velocity pattern, with an initial decrease during the backswing, a rapid increase toward ball contact, and a gradual decrease during follow-through; however, velocity was generally higher in BFFB, particularly from BV to VM (Figure 4).

### 3.2. Phase Duration Characteristics of Racket

**Figure 5:**
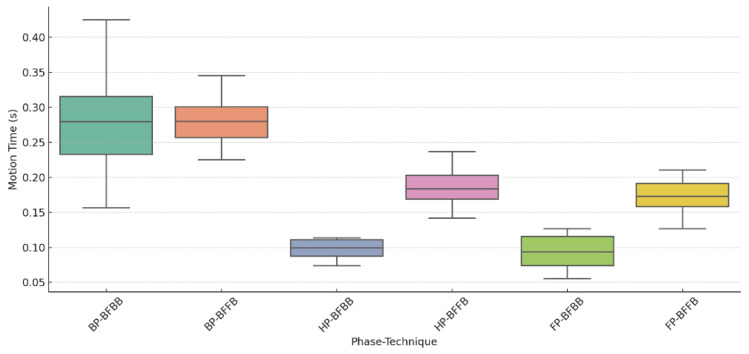
Distribution of racket phase durations during BP, HP, and FP in BFBB and BFFB.

Table 3 and Figure 5 show racket phase durations across the stroke cycle, including the backward phase (BP), hitting phase (HP), follow-through phase (FP), the combined HP + FP interval, and the entire phase (EP). Differences between BFBB and BFFB were observed mainly in the later phases of stroke execution. No significant difference in phase duration was observed between the two stroke combinations during BP (*p* = 0.962). In contrast, longer phase durations were observed in BFFB during HP and FP (both *p* < 0.001). When HP and FP were considered together, the combined duration remained greater in BFFB (*p* < 0.001). EP was also longer in BFFB than in BFBB (*p* < 0.001). Phase-duration distributions for BFBB and BFFB during BP, whereas the distributions for HP and FP were shifted toward longer durations in BFFB (Figure 5).

### 3.3. Spatial Displacement Characteristics of Racket

**Figure 6:**
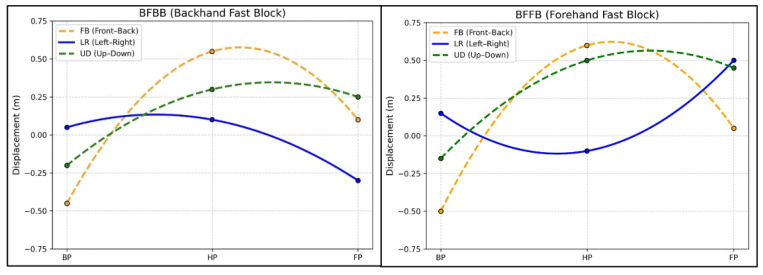
Directional racket displacement curves for BFBB and BFFB across BP, HP, and FP.

Table 4 and Figure 6 show phase-specific directional displacement of the racket during the backward phase (BP), hitting phase (HP), follow-through phase (FP), and across the total stroke cycle (TD). Differences between BFBB and BFFB were observed across stroke phases and spatial directions. During BP, significant differences were observed in the left–right (*p* = 0.003), front–back (*p* < 0.001), and up–down (*p* = 0.012) directions. During HP, displacement also differed significantly between the two stroke combinations in all three directions (all *p* < 0.001). A similar pattern was present during FP, where all three directional displacements remained significantly different, with the largest difference found in the left–right direction. Across TD, displacement also differed significantly between BFBB and BFFB in all three directions (all *p* < 0.001). Distinct directional displacement patterns were observed in BFBB and BFFB across BP, HP, and FP, with a larger spatial range in BFFB, particularly during HP and FP (Figure 6).

## 4. Discussions

Previous biomechanical research in table tennis has largely focused on the kinematic characteristics of isolated strokes, such as forehand topspin and service actions, whereas the movement organization involved in linking successive strokes has received relatively limited attention. Within this context, the present study examined how racket kinematics differ between two commonly used follow-up combinations after a backhand flick, namely a backhand fast block (BFBB) and a forehand fast block (BFFB). By considering racket velocity, temporal structure, and three-dimensional displacement together, the results showed that these two combinations were associated with clearly different kinematic profiles. Specifically, the transition into a forehand fast block was characterized by higher racket velocity, longer phase durations, and a broader spatial range, whereas the backhand follow-up showed a more compact movement pattern. These findings extend current kinematic descriptions of table tennis technique by showing how racket motion is reorganized when consecutive actions must be executed rapidly following a backhand flick.

### 4.1. Racket Velocity Dynamics

Racket velocity is an important kinematic variable in table tennis and has been widely examined in relation to stroke execution. In the present study, racket velocity was consistently higher in the backhand flick–forehand fast block combination (BFFB) than in the backhand flick–backhand fast block combination (BFBB) at several characteristic moments, particularly at the end of the backswing and around ball contact. These results suggest that the forehand follow-up was associated with a higher racket velocity than the backhand follow-up during the stroke transition sequence.

Previous biomechanical studies have shown that racket velocity plays an important role in isolated forehand and backhand strokes [25,27]. However, most existing work has focused on single-stroke execution and has provided limited insight into how velocity profiles are reorganized when strokes are performed consecutively under the time constraints of match play. In this context, the present findings extend previous work by showing that velocity-related differences remain evident when different subsequent strokes follow the backhand flick.

The higher racket velocities observed in BFFB, together with its larger displacement and longer phase durations, suggest a more extended kinematic pattern during the forehand follow-up. This interpretation is broadly consistent with previous descriptions of forehand actions as involving larger movement amplitudes than backhand actions [28]. However, the present study did not directly measure joint or segmental motion, so the whole-body mechanisms underlying these velocity differences remain to be clarified. By contrast, BFBB was associated with a more compact kinematic pattern following the initial flick, reflected in lower racket velocity and reduced spatial excursion. The present findings suggest that racket velocity is not only stroke-specific but also sequence-dependent when a backhand flick is followed by different subsequent strokes.

### 4.2. Temporal Organization of Stroke Phases

Across the two stroke combinations, the overall racket motion time differed noticeably. BFFB required more time to complete than BFBB, with the temporal difference emerging mainly after the stroke entered the later phases of execution. By contrast, BFBB was completed within a shorter time window, reflecting a more compact temporal pattern following the flick.

Longer phase durations for forehand actions have also been reported in previous analyses of isolated strokes [29,30]. A similar tendency was observed in the present data, where timing differences remained evident even when the strokes were performed as part of a rapid sequence rather than in isolation. In this context, overall racket timing appeared to differ according to the type of follow-up stroke, with the forehand transition in BFFB requiring a longer temporal window than the backhand transition in BFBB.

The distributional characteristics shown in Figure 5 further illustrate the temporal differences between the two combinations. During the backward phase, phase durations clustered closely in both conditions, with comparable medians and limited spread. In contrast, the hitting and follow-through phase showed a wider distribution in BFFB, whereas BFBB remained more tightly distributed around shorter durations. This pattern indicates that the temporal difference between the two combinations was concentrated mainly in the later phases of execution.

### 4.3. Spatial Displacement and Movement Patterns

Spatial displacement differed clearly between the two stroke combinations during the transition from the backhand flick to the subsequent stroke. During the backward phase, BFFB showed greater displacement in the front–back and up–down directions than BFBB, reflecting a more extended preparatory racket movement. This pattern is broadly consistent with previous descriptions of forehand actions as involving larger movement amplitudes [8,31].

During the hitting and follow-through phases, BFFB also showed greater displacement in all three spatial directions, suggesting a more extensive movement pathway during the forehand follow-up. The larger lateral and vertical excursions observed during the follow-through phase indicate that the follow-through in BFFB occurred over a broader spatial range, whereas BFBB remained more compact. This contrast is consistent with previous descriptions of the backhand fast block as involving a shorter swing path and faster completion close to the table [19].

Further insight into these spatial differences is provided by the three-dimensional trajectories shown in Figure 6. In the mediolateral direction, BFFB showed a broader displacement path than BFBB, whereas BFBB remained more compact following the initial flick. Such differences in trajectory direction likely reflect differences in how the racket is repositioned during stroke transition. The relationship between these racket-level patterns and whole-body coordination remains to be examined in future studies.

Across the full stroke cycle, BFFB involved greater overall displacement than BFBB, indicating a larger spatial range across the stroke sequence. By contrast, BFBB was performed within a smaller spatial range, consistent with a more compact movement pattern. These findings suggest that the two follow-up strokes were associated with different spatial organizations during the post-flick transition.

### 4.4. Implications for Training and Performance

From a training and performance perspective, the present findings suggest that BFBB and BFFB represent two different transition patterns after the backhand flick. BFBB was associated with a more compact spatial–temporal organization, whereas BFFB showed a more extended follow-up pattern with higher racket velocity and a larger movement range. These differences may be relevant when considering the demands of different stroke-transition situations in table tennis, a sport in which actions are performed under severe time constraints and rapid perception–action coupling is required [32].

The compact pattern observed in BFBB may be more suitable in situations where the follow-up stroke must be completed within a limited temporal and spatial window after the flick, especially in close-to-table play. By contrast, the more extended pattern observed in BFFB may be more relevant in situations where a fuller forehand continuation can be organized, and higher racket velocity can be produced. In this sense, the two combinations reflect not only different technical forms but also different transition demands after the backhand flick.

An important practical implication of the present study is that it shifts attention from isolated strokes to stroke-transition sequences. Recent work on combination strokes in table tennis has likewise emphasized that compound technique reflects real game situations more closely than single-stroke analysis and can provide information that is useful for coaches and athletes [26]. In this context, the present findings may inform sequence-specific training by distinguishing between more compact and more extended post-flick transition patterns at the racket level. Their practical value lies less in prescribing specific drills than in providing a kinematic reference for understanding how different follow-up strokes are organized under rapid sequential conditions.

### 4.5. Limitations and Future Directions for Research

Although this study provides meaningful insight into the kinematic differences between BFBB and BFFB stroke combinations, several limitations should be acknowledged. First, the sample consisted exclusively of high-level male athletes, which may limit the generalizability of the findings to other player populations. Previous research has shown that stroke mechanics and coordination patterns may differ by sex and skill level [18,19,24]. Future studies should include female players, athletes of different competitive levels, and individuals using different grip styles to determine whether the observed racket kinematic patterns are consistent across a broader range of players.

Second, this study focused exclusively on racket-level kinematic variables, including displacement, velocity, trajectory, and execution timing. Although racket kinematics reflect stroke execution and performance outcomes, they do not provide direct information on the underlying force production, joint loading, or muscle activation responsible for these movement patterns [26,33]. Integrating additional measurements such as joint kinetics, ground reaction forces, and surface electromyography would provide a more comprehensive understanding of the neuromuscular and mechanical factors contributing to stroke transitions and performance efficiency.

In addition, this analysis did not include detailed measurements of upper limb or trunk segment kinematics. While racket trajectory reflects the outcome of coordinated body motion, it does not fully capture the specific contributions of individual joints or joint coordination patterns. Previous studies have shown that the motion of the wrist, elbow, shoulder, and trunk contributes to racket velocity and stroke mechanics [24,30]. Future research integrating joint kinematics with racket-level measurements would help clarify the biomechanical basis of different stroke-transition strategies.

Finally, the standardized stroke sequences performed under laboratory conditions may not fully reflect the dynamic and unpredictable nature of real match conditions. In competitive situations, stroke execution is influenced by opponent behavior, ball variability, and time constraints, which can affect racket kinematics and movement coordination [34]. Future research may address this limitation by applying computer vision-based motion analysis to match video data, allowing accurate extraction of racket motion and joint kinematics during competition. Markerless motion capture and pose estimation techniques have demonstrated the ability to accurately capture biomechanical variables from video recordings and provide a practical approach for biomechanics analysis outside laboratory environments [35,36].

## 5. Conclusions

This study compared the racket-level kinematic characteristics of two common stroke transition sequences in elite table tennis: a backhand flick followed by a backhand fast block (BFBB) and a backhand flick followed by a forehand fast block (BFFB). Clear differences were observed between the two combinations in racket velocity, phase duration, and spatial displacement. Relative to BFBB, BFFB was characterized by higher racket velocity, greater displacement, and longer execution time, whereas BFBB showed a more compact kinematic pattern with a shorter duration and a smaller spatial range. These findings suggest that, even when the initial action is the same, the subsequent follow-up stroke is associated with distinct racket movement organization during the transition sequence. The results may provide a racket-level kinematic reference for understanding stroke-transition combinations in table tennis, extending current biomechanical analysis beyond isolated strokes.

## Figures and Tables

**Table 1:** The participants’ characteristics information (mean ± SD).

Population	Level	Age (Year)	Training Experience (Year)	Height (cm)	Weight (kg)
10	National Level I Athletes	22.50 ± 2.27	13.90 ± 1.79	177.4 ± 5.3	71.2 ± 9.1

**Table 2:** Comparison of racket velocity at characteristic moments between BFBB and BFFB (unit: m/s).

Characteristic Moments	BFBB Mean ± SD	BFFB Mean ± SD	Difference Mean ± SD	95%CI of Difference	Cohen’s dz	T	*p*
NP	2.18 ± 0.62	3.75 ± 1.19	−1.57 ± 0.71	[−2.08, −1.06]	−2.20	−6.964	<0.001
BE	1.55 ± 0.34	2.64 ± 1.14	−1.09 ± 0.92	[−1.74, −0.43]	−1.19	−3.750	0.005
BV	3.44 ± 0.92	6.21 ± 1.05	−2.77 ± 1.11	[−3.57, −1.98]	−2.49	−7.888	<0.001
CP	13.03 ± 1.52	14.24 ± 1.43	−1.21 ± 0.80	[−1.78, −0.64]	−1.52	−4.796	<0.001
VM	13.38 ± 1.40	14.49 ± 1.32	−1.11 ± 0.72	[−1.62, −0.60]	−1.54	−4.882	<0.001
PE	1.07 ± 0.44	0.78 ± 0.21	0.29 ± 0.46	[−0.04, 0.62]	0.63	1.981	0.079

Note: Data are presented as mean ± SD (*n* = 10). Difference, 95% CI, and Cohen’s dz are based on the paired difference (BFBB − BFFB). Statistical significance was evaluated using the Holm–Bonferroni correction within the family of velocity comparisons. BFBB, backhand flick following backhand fast block; BFFB, backhand flick following forehand fast block. NP, natural position; BE, end of the backswing; BV, velocity maximum of backswing; CP, point of contact; VM, velocity maximum; PE, end of the swing position.

**Table 3:** Comparison of racket phase durations between BFBB and BFFB (unit: s).

Phase	BFBB Mean ± SD	BFFB Mean ± SD	Difference Mean ± SD	95%CI of Difference	Cohen’s dz	T	*p*
BP	0.28 ± 0.08	0.28 ± 0.05	0.00 ± 0.09	[−0.06, 0.06]	0.02	0.049	0.962
HP	0.10 ± 0.01	0.19 ± 0.03	−0.09 ± 0.03	[−0.11, −0.07]	−2.95	−9.318	<0.001
FP	0.09 ± 0.03	0.17 ± 0.03	−0.08 ± 0.03	[−0.10, −0.06]	−2.89	−9.133	<0.001
HP + FP	0.19 ± 0.02	0.36 ± 0.04	−0.17 ± 0.04	[−0.19, −0.14]	−4.13	−13.06	<0.001
EP	0.47 ± 0.08	0.63 ± 0.06	−0.16 ± 0.09	[−0.23, −0.10]	−1.86	−5.872	<0.001

Note: Data are presented as mean ± SD (*n* = 10). Difference, 95% CI, and Cohen’s dz are based on the paired difference (BFBB − BFFB). Statistical significance was evaluated using the Holm–Bonferroni correction within the family of phase-duration comparisons. BFBB, backhand flick following backhand fast block; BFFB, backhand flick following forehand fast block. BP, backward phase; HP, hitting phase; FP, follow-through phase; EP, entire phase.

**Table 4:** Comparison of phase-specific directional racket displacement between BFBB and BFFB (unit: m).

Phase Direction		BFBB Mean ± SD	BFFB Mean ± SD	Difference Mean ± SD	95%CI of Difference	Cohen’s dz	T	*p*
BP	LR	0.07 ± 0.10	−0.40 ± 0.33	0.47 ± 0.37	[0.20, 0.73]	1.25	3.967	0.003
	FB	−0.50 ± 0.12	−0.97 ± 0.21	0.47 ± 0.22	[0.32, 0.63]	2.16	6.827	<0.001
	UD	−0.27 ± 0.08	−0.40 ± 0.18	0.13 ± 0.13	[0.04, 0.23]	1.00	3.156	0.012
HP	LR	0.10 ± 0.09	−0.39 ± 0.18	0.49 ± 0.24	[0.32, 0.66]	2.08	6.579	<0.001
	FB	0.65 ± 0.07	0.87 ± 0.15	−0.22 ± 0.14	[−0.32, −0.12]	−1.58	−4.980	<0.001
	UD	0.30 ± 0.06	0.65 ± 0.13	−0.35 ± 0.11	[−0.43, −0.27]	−3.14	−9.927	<0.001
FP	LR	−0.47 ± 0.09	0.86 ± 0.10	−1.33 ± 0.16	[−1.45, −1.21]	−8.15	−25.771	<0.001
	FB	0.08 ± 0.07	−0.04 ± 0.16	0.12 ± 0.13	[0.02, 0.21]	0.86	2.712	0.024
	UD	0.22 ± 0.07	0.40 ± 0.13	−0.18 ± 0.08	[−0.24, −0.12]	−2.16	−6.842	<0.001
TD	LR	0.95 ± 0.20	1.92 ± 0.25	−0.97 ± 0.29	[−1.17, −0.76]	−3.39	−10.704	<0.001
	FB	1.36 ± 0.16	2.43 ± 0.21	−1.07 ± 0.22	[−1.22, −0.91]	−4.90	−15.503	<0.001
	UD	0.90 ± 0.09	1.68 ± 0.22	−0.78 ± 0.19	[−0.92, −0.64]	−4.01	−12.689	<0.001

Note: Data are presented as mean ± SD (*n* = 10). Difference, 95% CI, and Cohen’s dz are based on the paired difference (BFBB − BFFB). Statistical significance was evaluated using the Holm–Bonferroni correction within the family of velocity comparisons. BFBB, backhand flick following backhand fast block; BFFB, backhand flick following forehand fast block. BP, backward phase; HP, hitting phase; FP, follow-through phase; TD, total distance. LR, left & right; FB, front & back; UD, up & down.

## Data Availability

The original contributions presented in this study are included in the article. Further inquiries can be directed to the corresponding authors.
